# Regional distribution of *Mycobacterium tuberculosis* infection and resistance to rifampicin and isoniazid as determined by high-resolution melt analysis

**DOI:** 10.1186/s12879-022-07792-7

**Published:** 2022-10-31

**Authors:** Zhenzhen Wang, Tengfei Guo, Tao Jiang, Zhanqin Zhao, Xiangyang Zu, Long li, Qing Zhang, Yi Hou, Kena Song, Yun Xue

**Affiliations:** 1 The First Affiliated Hospital and Clinical Medical College, Henan University of Science and Technology, 471000 Luo Yang, China; 2 School of Medical Technology and Engineering, Henan University of Science and Technology, Luo Yang, 471000 China; 3 Animal Science and Technology, Henan University of Science and Technology, Luo Yang, 471000 China; 4 Luoyang City CDC, Luo Yang, 471000 China

**Keywords:** Multidrug-resistant tuberculosis, Transmission, Molecular epidemiology, Resistance model

## Abstract

**Background:**

Identifying the transmission mode and resistance mechanism of *Mycobacterium tuberculosis* (MTB) is key to prevent disease transmission. However, there is a lack of regional data. Therefore, the aim of this study was to identify risk factors associated with the transmission of MTB and regional patterns of resistance to isoniazid (INH) and rifampicin (RFP), as well as the prevalence of multidrug-resistant tuberculosis (MDR-TB).

**Methods:**

High-resolution melt (HRM) analysis was conducted using sputum, alveolar lavage fluid, and pleural fluid samples collected from 17,515 patients with suspected or confirmed MTB infection in the downtown area and nine counties of Luoyang City from 2019 to 2021.

**Results:**

Of the 17,515 patients, 82.6% resided in rural areas, and 96.0% appeared for an initial screening. The HRM positivity rate was 16.8%, with a higher rate in males than females (18.0% vs. 14.1%, *p* < 0.001). As expected, a positive sputum smear was correlated with a positive result for HRM analysis. By age, the highest rates of MTB infection occurred in males (22.9%) aged 26–30 years and females (28.1%) aged 21–25. The rates of resistance to RFP and INH and the incidence of MDR were higher in males than females (20.5% vs. 16.1%, *p* < 0.001, 15.9% vs. 12.0%, *p* < 0.001 and 12.9% vs. 10.2%, *p* < 0.001, respectively). The HRM positivity rate was much higher in previously treated patients than those newly diagnosed for MTB infection. Notably, males at the initial screening had significantly higher rates of HRM positive, INH resistance, RFP resistance, and MDR-TB than females (all, *p* < 0.05), but not those previously treated for MTB infection. The HRM positivity and drug resistance rates were much higher in the urban vs. rural population. By multivariate analyses, previous treatment, age < 51 years, residing in an urban area, and male sex were significantly and positively associated with drug resistance after adjusting for smear results and year of testing.

**Conclusion:**

Males were at higher risks for MTB infection and drug resistance, while a younger age was associated with MTB infection, resistance to INH and RFP, and MDR-TB. Further comprehensive monitoring of resistance patterns is needed to control the spread of MTB infection and manage drug resistance locally.

## Introduction

The incidence of *Mycobacterium tuberculosis* (MTB) infection is reportedly decreasing, although the actual number of cases could be far greater due to under-reporting and misdiagnosis [1]. Despite the long history of drug development, MTB infection continues as a public health concern. Notably, multidrug-resistant tuberculosis (MDR-TB) and rifampicin (RFP)-resistant TB are particularly tenacious due to the toxicity of anti-TB drugs, high mortality rate, and substantial economic burden [2]. The World Health Organization estimated that there were approximately 465,000 cases of drug-resistant tuberculosis (DR-TB) worldwide in 2019 and only about one-third of MDR-TB cases received appropriate treatment [1, 3]. Inappropriate treatment leads to the development of drug resistance and poor outcomes [4, 5]. Therefore, clarification of regional distributions and risk factors associated with the development of drug resistance could improve utilization of limited healthcare resources to effectively control the spread of MTB infection. Although phenotypic antibiotic susceptibility testing remains the current gold standard for diagnosis of DR-TB [6], this method is time-consuming due to the relatively long detection cycle. Therefore, molecular diagnostic techniques should be more widely applied in clinical practice for detection and resistance analysis for rapid and effective control of MTB infection. High-resolution melt (HRM) analysis [7] can be used to simultaneously analyze sensitivity to first- and second-line drugs as supplemental epidemiological data of regional MTB infection and drug resistance.

Drug resistance, pathogenic toxicity, inappropriate treatment, and rapid urbanization are all associated with the continued spread and treatment outcome of MTB infection [8–10]. Since transmission and resistance patterns vary geographically [11], incomplete surveillance data can hinder timely treatment of high-risk groups and assessment of drug resistance [12]. Although treatment history is considered an important risk factor, increasing numbers of patients are initially diagnosed with DR-TB, suggesting the need to increase monitoring of drug resistance [13, 14]. The incidence of MTB infection is regularly reported in many regions, complementing global surveillance and monitoring databases. However, data regarding transmission characteristics, molecular resistance patterns, and risk factors for DR-TB in in this region remain limited. Therefore, the aim of this study was to identify risk factors related to MTB transmission and drug resistance to provide theoretical support for local management of MTB infection in China.

## Materials and methods

From January 2019 to December 2021, HRM analysis was performed using sputum, pleural fluid, and bronchoalveolar lavage fluid samples collected from all patients with clinically confirmed or suspected MTB infection. The patients included those with clinical symptoms or latent infection, immunopathological features of MTB infection, imaging signs of infection, close contacts with excreting patients, strong positive tuberculin test reactions and history of MTB infection residing in Luoyang City and county districts (Yanshi, Luanchuan, Songxian, Ruyang, Luoning, Mengjin, Xin’an, Yiyang, and Yichuan).

Prior to analysis, duplicate data were excluded by name, sex, age, and region. Individuals aged < 15 and > 86 years were combined into a single age category, while all others were classified into 5-year age groups. Variables included the date of testing, HRM-positive results, INH resistance (INH-R), RFP resistance (RFP-R), multidrug resistant (MDR), sex, age, region (county-districts are uniformly classified as township areas), sputum smear results, and systematic recorded or self-reported treatment history. MDR-TB was defined as resistance to at least INH and RIF. The patients were classified according to the history of treatment. Newly diagnosed patients, those who have never received anti-MTB treatment, those undergoing standard anti-MTB treatment regimens and have not completed the course of treatment, those receiving irregular anti-MTB therapy for less than 1 month. Retreatment patients, those who received anti-MTB drugs in the past for one month or more, those failed or relapsed in the initial treatment [15, 16]. Initial resistance refers to a newly diagnosed case of DR-MTB. Acquired resistance refers to resistance in previously treated patients who received first-line drugs for at least 4 weeks. Patients newly diagnosed with MDR, as confirmed by HRM analysis, received drug-resistant or MDR-TB management regimen and were not included in the previously treated group. In this study, resistance to RFP was confirmed by the presence of the 81-bp core region (rifampicin-resistance-determining region) of the *rpoB* gene (codons 426–452 of MTB correspond to codons 507–533 of *Escherichia coli*) [17] and resistance to INH was confirmed by presence of the codon katG315, inhA promoter region (locus − 17 ~ -8 locus), AhpC promoter region (locus − 44 ~ -30 and − 15 ~ 3), and codon inhA94.

### Statistical analysis

The HRM analysis results, HRM-positive results, and rates of RFP-R, INH-R, and MDR-TB were stratified by sex for the newly diagnosed and previously treated groups, urban and rural groups, and sputum-positive and -negative groups. The HRM-positive groups, as well as the RFP-R, INH-R, and MDR-TB groups were then stratified by age to confirm the time pattern of DR-MTB infection. Meanwhile, the prevalence of specific mutations related to drug resistance was also assessed. Multivariate logistic regression models were created with all variables that may be associated with RFP-R, INH-R, and MDR-TB to assess potential correlations with other covariates. The odds ratio (OR) and 95% confidence interval (CI) were calculated. Pearson’s chi-square analysis was performed to identify significant differences in the detection and resistance rates of MTB under different conditions. A probability (*p*) value of < 0.05 was considered statistically significant. All analyses were performed using STATA/SE 15.1 software (StataCorp LLC, College Station, TX, USA).

## Results

The frequency of HRM analysis increased each year during the study period, from 3662 cases (2645 males, 1017 females) in 2019 to 7893 cases (5249 males, 2599 females) in 2021. From 2019 to 2021, HRM analysis was conducted with samples from 17,515 patients, which included 12,054 (68.8%) males and 5461 (31.2%) females. More than 95.0% were newly diagnosed patients, 82.6% resided in rural area, and only 12.3% of the sputum smears were positive for acid-fast bacilli. Of the 17,515 patients included for initial HRM analysis, 16.8% had positive results, with significantly more males than females (18.0% vs. 14.1%, *p* < 0.001), of which 19.3% were resistant to INH, 14.9% were resistant to RFP, and the incidence of MDR-TB was 12.2%. The HRM positivity rate was significantly higher in previously treated patients than newly diagnosed patients (51.5% vs. 15.3%, *p* < 0.001). Among the newly diagnosed patients, the HRM positivity rate was significantly higher in males than females (16.9% vs. 11.9%, *p* < 0.001), whereas among the previously treated patients, the HRM positivity rate was significantly higher in females than males (63.8% vs. 45.6%, *p* < 0.001). Moreover, the HRM positivity rate was significantly higher in patients residing in urban rather than rural areas (35.3% vs. 12.9%, *p* < 0.001), with significantly higher rates in males than females in both urban and rural areas (36.9% vs. 31.5%, *p* = 0.004; 13.9% vs. 10.6%, *p* < 0.001). The HRM positivity rate was significantly higher in patients with positive vs. negative sputum smear results (76.0% vs. 7.9%, *p* < 0.001), although there was no significant difference in the detection rate between sexes (Table 1).

**Fig. 1:**
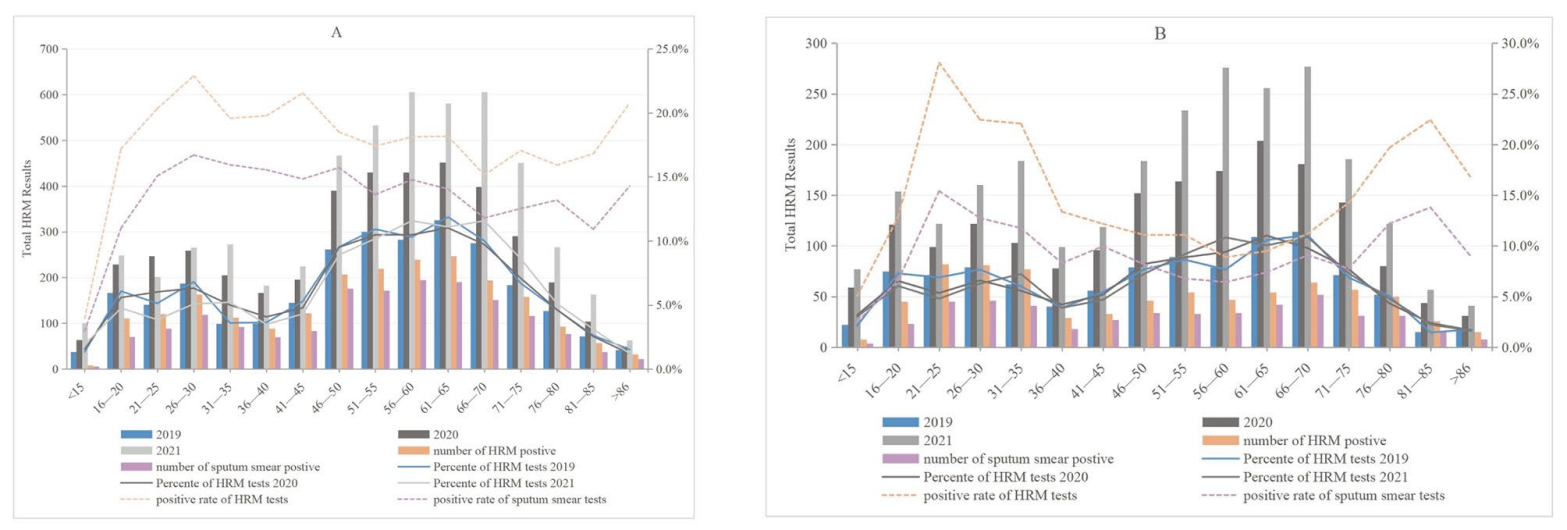
Number of HRM tests and positive results stratified by age category for males (A) and females (B). The annual count of HRM test results for each age classification is expressed in bars.The proportion of participating HRM tests and HRM-positive results , as well as the proportion of smears positive for each age category are indicated by lines (second y-axis)

Both males and females aged 51–70 years were more likely to undergo HRM analysis. HRM-positive males mainly appeared in 51–65 years old, accounting for 32.5%. In contrast, HRM-positive females mainly concentrated in 21–35 years old, with a proportion of 31.2% (Fig. 1 A and 1B, respectively). Among males, the HRM positivity rate peaked at 26–30 years to 22.9%, then basically stabilized, but remained above 15% in all age categories. In contrast, among females, the HRM positivity rate peaked at 21–25 years to 28.1%, then decreased rapidly to 13.4% at 36–40 years. The median (interquartile range, IQR) age of males who underwent HRM testing and had positive results were 55 (39–67) and 54 (37–65) years, respectively, while in females were 54 (IQR 33, 66) and 48 years (IQR 29, 67), respectively (data not shown).

**Fig. 2:**
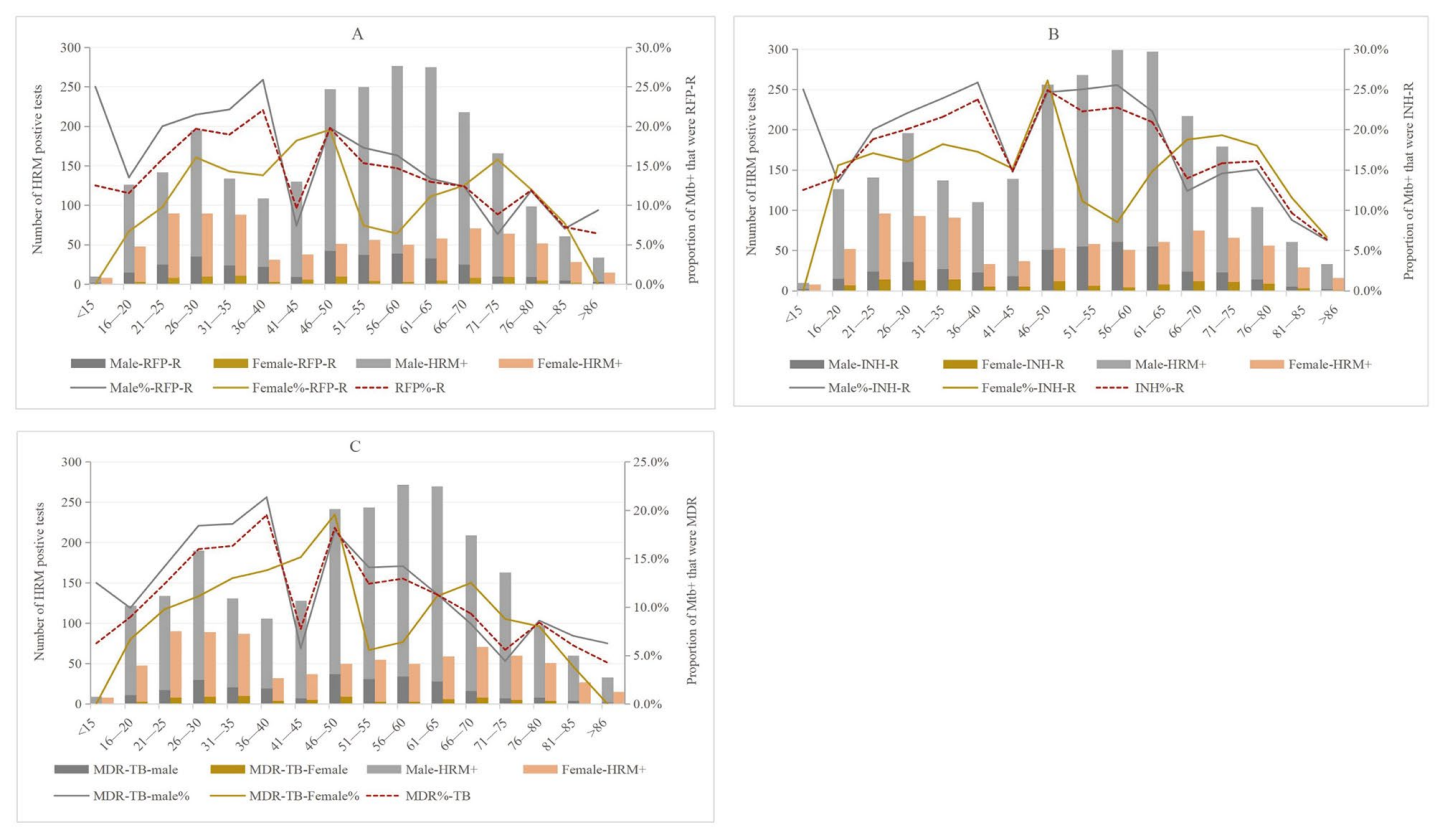
MTB positive results and the proportion of RFP-R(A), INH-R(B) and MDR(C) by age and gender category. The total number of MTB positive by age are expressed in bars, and the proportion for RFP-R, INH-R and MDR are expressed in lines (second y-axis). RFP-R rifampicin-resistant, INH-R isoniazid-resistant

During study period, the number of HRM-positive cases increased from 2019 to 2020 and to 2021, although the HRM positivity rate decreased in both males (24.6%, 16.5%, and 15.9, respectively) and females (21.1%, 12.8%, and 12.2%, respectively). Similarly, although the number of INH-R, RFP-R, and MDR-TB cases had increased from 2019 to 2021, the overall incidence of drug resistance tended to decrease in both males and females.

**Table 1:** Population characteristics of the study stratified by sex

	Sex		*p*
	Males n (%)	Females n (%)	
HRM-Test result (%)	12,054(68.8)	5461(31.2)	/
HRM-Positive result (%)	2173(18.0)	768(14.1)	< 0.001
Treated			
New suspected cases (%)	8784(72.9)	4202(76.8)	/
New confirmed cases (%)	2790(23.1)	1030(18.9)	/
Previously treated cases (%)	480(4.0)	229(4.2)	/
Positive result of new cases (%) *	1954(16.9)	622(11.9)	< 0.001
Positive result of retreated cases (%)	219(45.6)	146(63.8)	< 0.001
Region			
Urban (%)	2153(17.9)	901(16.5)	/
Rural (%)	9901(82.1)	4560(83.5)	/
Positive result of urban (%)	795(36.9)	284(31.5)	0.004
Positive result of rural (%)	1378(13.9)	484(10.6)	< 0.001
Sputum smear			
Negative (%)	9173(76.1)	4301(78.8)	/
Positive (%)	1646(13.7)	518(9.5)	/
Unknown (%)	1235(10.2)	642(11.8)	/
Positive result of smear+ (%)	1272(77.3)	392(75.1)	0.360
Positive result of smear- (%)	752(8.2)	314(7.3)	0.072
Test year			
2019	2645	1017	/
2020	4115	1845	/
2021	5294	2599	/

* The data includes new suspected cases and new confirmed cases

When stratified by age, the highest rates of RFP-R, INH-R, and MDR occurred in males aged 36–40 years and females aged 46–50 years. Among males, 25.9% were resistant to RFP, 25.8% were resistant to INH, and 21.3% were diagnosed with MDR-TB. Among females, 19.6% were resistant to RFP, 26.1% were resistant to INH, and 18.2% were diagnosed with MDR-TB. As shown in Fig. 2 A, the RFP-R rate was lower in males than females at the ages of 41–45 and 71–75 years, but higher than in females in all other age groups. To identify factors responsible for this difference, analysis of the characteristics of HRM-positive populations in different age groups found that the proportions of urban-dwelling males aged 41–45 and 71–75 years (34.4% and 26.6%, respectively) were lower than the mean proportion of the urban-dwelling male population in each age range (36.9%), while the proportions of rural-dwelling males (65.6% and 73.4%, respectively) were higher than the mean proportion of each age range (63.1%). Meanwhile, the proportions of previously treated females aged 41–45 and 71–75 years (18.2% and 24.6%, respectively) were higher than the mean proportions of previously treated females at each age range (16.7%), while the proportions of newly diagnosed females (81.8% and 75.4%, respectively) were lower than the mean proportions of newly diagnosed females at each age range (83.3%). The detection rates of MDR-TB and INH-R among all age groups showed a trend of drug resistance consistent with RFP-R (Fig. 2B C). The data for drug resistance in patients aged < 15 and > 85 years in this study were limited and, thus, will be included in future studies

The rates of RFP-R, INH-R, and MDR-TB were 18.9%, 14.0% and 11.4%, respectively, among newly diagnosed patients, 22.7%, 20.8%, and 17.5% among previously treated patients, 23.2%, 18.9%, and 15.5% among patients residing in urban areas, 17.1%, 12.5%, and 9.4% among patients residing in rural areas, 20.4%, 16.3%, and 13.0% among patients with positive sputum results, and 18.1%, 12.4%, and 10.6% among patients with negative sputum results. RFP-R, INH-R, and MDR-TB were related to sex, age, previous treatment of TB, and region (Table 2)

**Table 2:** Resistance of RFP, INH and MDR under different factors by sex

	Sex		*p*
	Males n (%)	Females n (%)	
INH-Resistant (%)	445(20.5)	124(16.1)	0.009
RFP-Resistant (%)	345(15.9)	92(12.0)	0.009
MDR-TB (%)	280(12.9)	78(10.2)	0.047
New patients			
INH-Resistant (%)	396(20.3)	90(14.5)	< 0.001
RFP-Resistant (%)	299(15.3)	62(10.0)	< 0.001
MDR-TB (%)	243(12.4)	51(8.2)	0.004
Previously treated			
INH-Resistant (%)	49(22.4)	34(24.0)	0.838
RFP-Resistant (%)	46(21.0)	30(20.5)	0.916
MDR-TB (%)	37(16.9)	27(18.5)	0.694
Smear+ (%)			
INH-Resistant (%)	277(21.8)	62(15.8)	0.010
RFP-Resistant (%)	226(17.8)	46(11.7)	0.005
MDR-TB (%)	180(14.2)	36(9.2)	0.011
Smear- (%)			
INH-Resistant (%)	139(18.5)	54(17.2)	0.619
RFP-Resistant (%)	94(12.5)	38(12.1)	0.875
MDR-TB (%)	78(10.4)	35(11.1)	0.708
Smear unknow (%)			
INH-Resistant (%)	29(19.5)	8(12.9)	0.254
RFP-Resistant (%)	25(16.8)	8(12.9)	0.480
MDR-TB (%)	22(14.8)	7(11.3)	0.504
Urban			
INH-Resistant (%)	197(24.8)	53(18.7)	0.036
RFP-Resistant (%)	162(20.4)	42(14.8)	0.039
MDR-TB (%)	134(16.9)	33(11.6)	0.036
Rural			
INH-Resistant (%)	248(18.0)	71(14.7)	0.095
RFP-Resistant (%)	183(13.3)	50(10.3)	0.012
MDR-TB (%)	146(10.6)	45(9.3)	0.418
HRM-positive results of Test Year			
2019	651(24.6)	215(21.1)	0.027
2020	679(16.5)	237(12.8)	< 0.001
2021	843(15.9)	316(12.2)	< 0.001

**Fig. 3:**
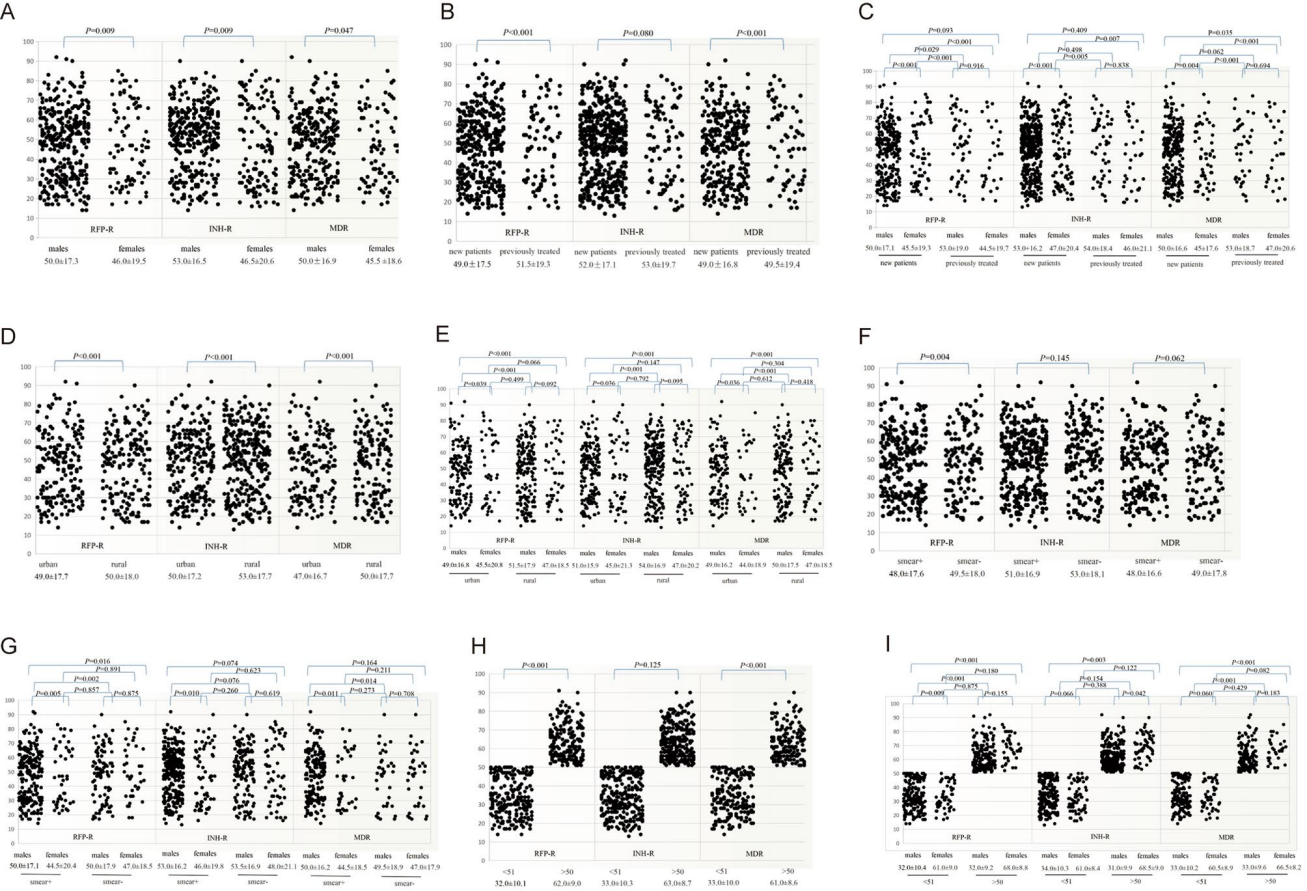
Represented correlations among patient age, treatment history, region, smear, and gender by scatter plots. The dots indicate the age of patients of DR-TB and the median age is indicated below each variable. A B D F H indicate differences in resistance to INH, RFP and MDR-TB in the context of different gender, treatment history, regional distribution, sputum smear results, age groups greater than 50 years or less than 51 years, respectively. C E G I indicate differences in detection to RFP-R, INH-R and MDR-TB by sex in the context of different TB treatment history, region, sputum smear results, less than 51 or older than 50 years

Scatter plots were created with age plotted along the y-axis and different variables along the x-axis (Fig. 3). As indicated by the scatter plots presented in Fig. 3 A, there were significant differences between males and females in the incidences of INH-R (20.5% vs. 16.1%, χ^2^ = 6.827, *p* = 0.009), RFP-R (15.9% vs. 12.0%, χ^2^ = 6.814, *p* = 0.009), and MDR (12.9% vs. 10.2%, χ^2^ = 3.953, *p* = 0.047). As shown in Fig. 3B, there were significant differences in the detection rates of RFP-R and MDR-TB between the previously treated and newly diagnosed groups (all, *p* < 0.001), but not INH-R (*p* = 0.080). Hence, Pearson’s chi-square analysis was also performed to assess difference in the rates of RFP-R, INH-R, and MDR-TB between males and females in consideration of previous treatment or not for MTB infection. The results revealed statistically significant differences in the rates of INH-R, RFP-R, and MDR-TB between newly diagnosed males and females, but not those previously treated for MTB infection, as shown in Fig. 3 C. Also, the differences between the detection rates of INH-R, RFP-R and MDR-TB in the retreatment females and those in newly diagnosed were statistically significant (*p* = 0.007, *p* < 0.001 and *p* < 0.001, respectively), whereas only RFP-R was significantly different between males previously treated and newly diagnosed (*p* = 0.029). The results of Pearson’s chi-square analysis revealed significant differences in the rates of RFP-R, INH-R, and MDR-TB between patients residing in urban vs. rural areas (all, *p* < 0.001). There were also significant differences in resistance rates between males and females residing in urban (*p =* 0.039, 0.036, and 0.036, respectively), but not rural areas (*p =* 0.092, 0.095, and 0.418, respectively) (Fig. 3D and E, respectively). As indicated in Fig. 3 F, only RFP-R was statistically significant between positive and negative sputum smears (*p* = 0.004). Also, there were significant differences in the rates of INH-R, RFP-R, and MDR-TB between males and females with positive sputum results (*p* = 0.010, 0.005, and 0.011, respectively). As shown in Fig. 3G, there was a significant difference in the rate of RFP-R between males with positive sputum results and females with negative sputum results (*p* = 0.016). Analysis of drug resistance rates among different age groups showed that the incidences of RFP-R and MDR-TB, but not INH-R, were significantly higher in patients aged < 51 vs. >50 years (*p* < 0.001, *p* < 0.001, and *p =* 0.125, respectively), while the difference in INH resistance rate can be reflected between males aged < 51 years and females aged > 50 year (*p* = 0.003), and between males and females aged > 50 years (*p* = 0.042), (Fig. 3 H and 3I, respectively).

**Fig. 4:**
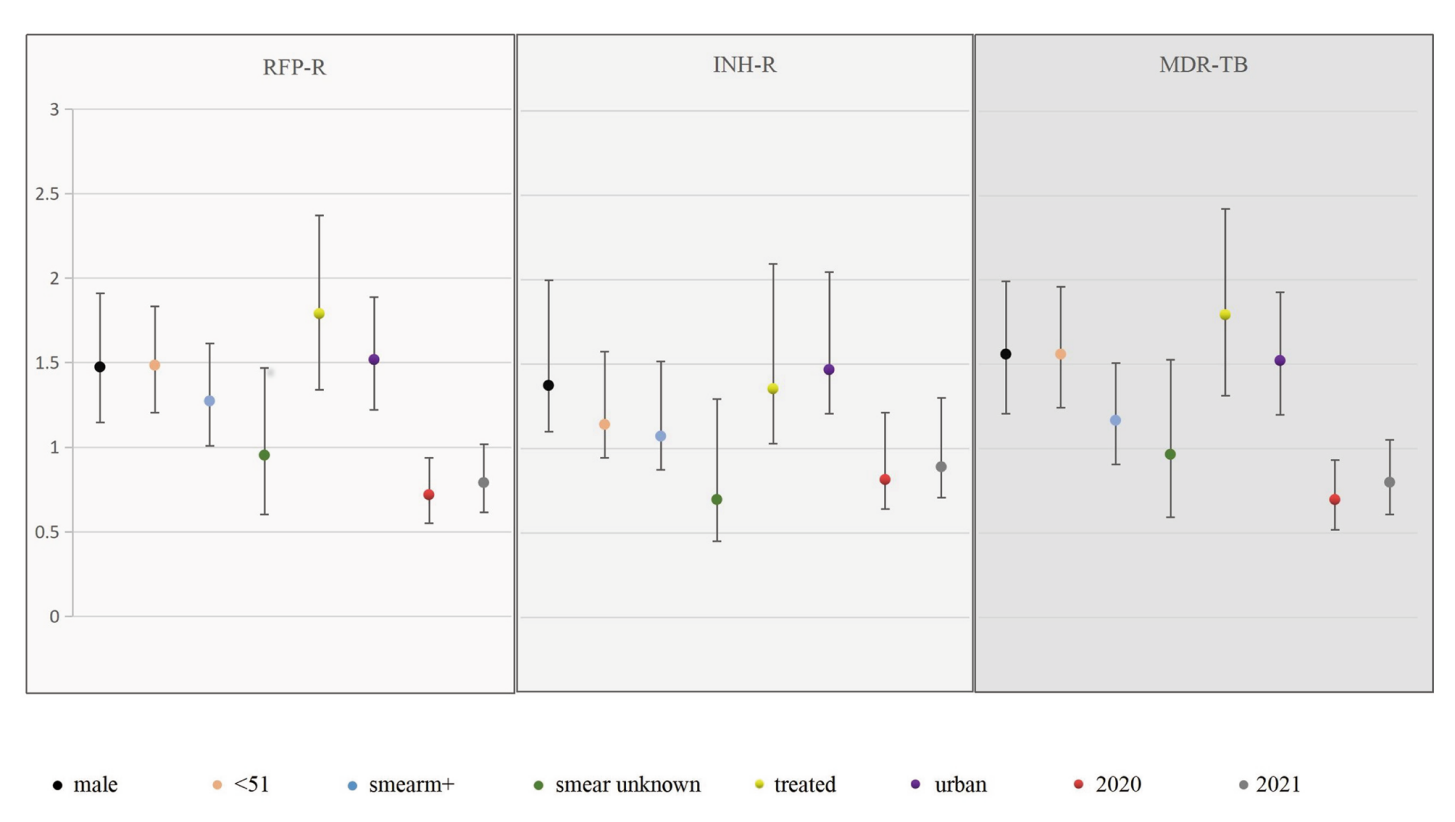
Graphic expression of the final model of multivariate logistic regression for factors with RFP-R, INH-R, MDR in cases of HRM-positive tuberculosis. (2941 cases of HRM positive from 2019 to 2021)

Multivariate logistic regression analysis was used to determine whether sex, age, previous TB treatment, region, sputum smear results, and year of testing were associated with the incidence of INH-R, RFP-R, and MDR-TB. The results obtained with an adjusted model revealed that male, age < 51 years, urban area, and previous treatment for TB were positively associated with an increased risk of MTB resistance (Fig. 4). After taking into account the sputum results and year of detection, the risks of RFP-R, INH-R, and MDR-TB were increased in males by 1.47-fold (95% CI = 1.15–1.91), 1.37-fold (95% CI = 1.10–1.73), and 1.56-fold (95% CI = 1.24–1.96), respectively, as compared to females, and were increased by 1.79-fold (95% CI = 1.33–2.37), 1.35-fold (95% CI = 1.03–1.77), and 1.79-fold (95% CI = 1.31–2.42) in patients with a history of MTB infection as compared to those newly diagnosed. In addition, risks of drug resistance were increased by 1.52-fold (95% CI = 1.22–1.89), 1.47-fold (95% CI = 1.21–1.78), and 1.52-fold (95% CI = 1.20–1.93) in patients residing in urban vs. rural areas, and by 1.49-fold (95% CI = 1.20–1.83), 1.14-fold (95% CI = 0.95–1.38), and 1.56-fold (95% CI = 1.24–1.96) in patients aged < 51 vs. >50 years (Table 3). See End of Text.

**Table 3:** Final model of multivariate logistic regression for RFP-R, INH-R and MDR-TB associated with sex, age, history of previous treatment, region, smear and year of HRM assay. Total HRM positive n = 2,941

	RFP-R					INH-R					MDR-TB				
	n	%	Adjusted OR	(CI95%)	p*	n	%	Adjusted OR	(CI95%)	p*	n	%	Adjusted OR	(CI95%)	p*
Sex															
Female	92	12.0	reference			124	16.1	reference			78	10.2	reference		
Male	345	15.9	1.47	1.15,1.91	< 0.001	445	20.5	1.37	1.10,1.73	< 0.001	280	12.9	1.42	1.21,1.99	0.012
Age															
> 50	200	12.5	reference			293	18.3				160	10.0			
< 51	237	17.7	1.49	1.20,1.83	< 0.001	276	20.6	1.14	0.95,1.38	0.166	198	14.8	1.56	1.24,196	< 0.021
Smear															
Negative	132	12.4	reference			193	18.1	reference			113	10.6	reference		
Positive	272	16.3	1.27	1.01,1.61	0.045	339	20.4	1.07	0.87,1.32	0.505	216	13	1.17	0.91,1.51	0.235
Unknown	33	15.6	0.95	0.60,1.47	0.840	37	17.5	0.70	0.45,1.05	0.093	29	13.7	0.97	0.59,1.53	0.884
Previous treatment															
New patients	361	14.0	reference			486	18.9	reference			294	11.4	reference		
Retreatment Cases	76	20.8	1.79	1.33,2.37	< 0.001	83	22.7	1.35	1.03,1.77	0.029	64	17.5	1.79	1.31,2.42	< 0.001
Region															
Rural	233	12.5	reference			319	17.1	reference			191	10.3	reference		
Urban	204	18.9	1.52	1.22,1.89	< 0.001	250	23.2	1.47	1.21,1.78	< 0.001	167	15.5	1.52	1.20,1.93	< 0.001
Year of test															
2019	147	17.0	reference			177	20.4	reference			122	14.1	reference		
2020	122	13.3	0.72	0.55,0.94	0.015	166	18.1	0.82	0.64,1.04	0.098	98	10.7	0.70	0.52,0.93	0.015
2021	168	14.5	0.79	0.61,1.02	0.068	226	19.5	0.89	0.71,1.12	0.321	138	11.9	0.80	0.61,1.05	0.109

OR odds ratio, CI confidence interval, * Significance level = 0.05

**Fig. 5:**
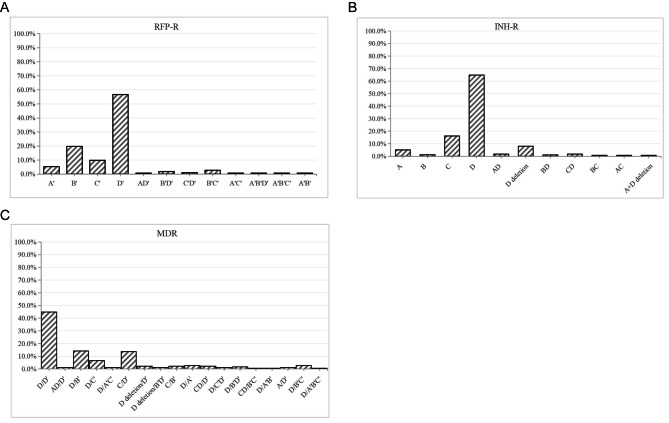
Prevalence of resistance-conferring mutations in genes of MTB strains. The gene coverage region and mutation points is INH A AhpC promoter region (-44 ~ -30 and-15 ~ 3 sites), B inhA94 codon, C inhA promoter region (-17 ~ -8 site), D KatG315 codon. RFP(covering the *rpoB* gene) A’ 507–512 codon, B’ 521-528codon, C’ 513-520codon, D’ 529-533codon

Among the gene mutations associated with RFP-R, 91.5% of cases had one mutation, 7.6% had two, and 1.1% had three. The sites with the highest mutation rates of the *rpoB* gene were codons 529–533 and 521–528, which accounted for 56.7% and 19.7% of all mutations, respectively. The most common double mutation occurred at codons 521–528 and 513–520 followed by codons 521–528 and 529–533, which accounted for 36.4% and 24.2%, respectively. Meanwhile, the most common triple mutations involved codons 507–512, 521–528, and 513–520 (three cases), followed by codons 507–512, 521–528, and 529–533 (two cases). Among the mutations associated with INH-R, single and double mutations were identified in 95.4% and 4.6% of patients, respectively. The most common point mutations involved katG315 and the inhA promoter region (-17 ~ -8 site), which accounted for 88.5% and 16.1% of all mutation types, respectively, of which katG315 mutations accounted for 64.9% and katG315 deletions for 8.1%. The AhpC promoter region (-44 ~ -30 and − 15 ~ 3 sites) or the inhA promoter region (-17 ~ -8 site) combined with katG315 accounted for the highest double mutation rates of 36.8% and 36.8%, respectively. The molecular patterns associated with MDR-TB were mutations to katG315 combined with codon 529–533 or 521–528 and the inhA promoter region (-17 ~ -8 site) combined with codon 529–533, which accounted for 44.8%, 14.2%, and 13.7% of all mutation patterns, respectively (Fig. 5).

## Discussion

Prevention and control of MTB infection should be prioritized among high-risk groups [18]. Actually, before HRM testing, clinical screening was performed to rule out low-risk groups of TB infection. The vast majority (95%) of these populations were newly diagnosed patients and from rural areas (82.6%), indicating that individuals residing in rural areas are at higher risk for MTB infection.

HRM analysis is a highly reliable method for identification of MTB infection and highly accurate for detection of drug resistance, particularly to first-line anti-TB drugs [19]. The accuracy of HRM analysis is reportedly greater than 80.0% for resistance to first-line anti-TB drugs and more than 89.0% for second-line anti-TB drugs [20]. Therefore, this method is considered a viable option for rapid diagnosis of DR-TB. In the present study, the HRM positivity rate was higher in males than females (18.0% vs. 14.1%, respectively), indicating that males are at greater risk for MTB infection, which is consistent with previous studies conducted in other regions of China [21]. A possible explanation for this difference in susceptibility between sexes may be related to poorer lifestyle habits of males [22]. The positivity rate of HRM was much higher in groups with positive sputum smears and a history of TB treatment, indicating that positive results of HRM analysis may be closely related to the load of MTB. The HRM positivity rate was much lower in the rural vs. urban group (12.9% vs. 35.3%, respectively), possibly due to differences in healthcare availability in rural areas. Overcrowding in urban areas results in the rapid spread of MTB infection and the continued development of reporting networks and the addition of healthcare centers make the reporting of TB positive cases in urban areas more timely and comprehensive may explain why the detection rate is higher in urban vs. rural areas. In addition, the proportions of previously treated cases and positive sputum smears were lower in rural than urban areas (3.8% vs. 5.8% and 9.5% vs. 25.8%, respectively) (results not shown).

In this study, there were notable differences in the incidence of MTB infections among age groups. Although both males and females participated in HRM testing, most were aged 51–70 years, the highest rate of MTB infection occurred in males aged 26–30 years and females aged 21–25 years, suggesting that young adults might be more susceptible to MTB infection than previously reported, which may be related to hormone-mediated immune responses, increased risk of lung disease, and non-standardized treatments [23, 24]. In addition, young adults are more likely to gather in groups, thereby facilitating the spread of MTB infection [25]. Although young adults are generally considered to be at low risk for MTB infection, additional testing of this group would help to clarify the risk factors associated with the transmission of MTB [26]. Notably, about 2.5 times more males aged 51–65 years undergo HRM testing as compared to females was a main reason why the males MTB positive groups were main at this ages. The rate of MTB infection in males remained greater than 15% in all other age groups, shows, compared with females, the tuberculosis in males throughout the life course is all the focus of management.

Overall, the ratio of MTB-positive males to females was 2.8, while the ratios of males to females with INH-R, RFP-R, and MDR-TB were 3.6, 3.8, and 3.6, respectively, indicating that males were at greater risks for DR-TB not only due to the high ratio of MTB infection in males than females, consistent with a previous study conducted in China [27]. Between 2019 and 2021, the HRM positivity rate as well as the rates of INH-R, RFP-R, MDR tended to decrease, demonstrating that strict control strategies and management of confirmed cases of MTB infection helped to prevent the spread of DR-TB, as least to a certain extent. The decline in drug resistance also reflects the effectiveness of the control strategy recommended by the World Health Organization (i.e., “Directly Observed Treatment, Short-Course”).

Drug resistance was more common in patients previously treated for MTB infection than newly diagnosed cases. Initial drug resistance reflects the ability of local DR-TB to spread and highlights the need for prevention and control strategies. Among newly diagnosed patients, drug resistance tended to decrease over the study period, indicating successful management of primary DR-TB in Luoyang City from 2019 to 2021. However, the MDR and RFP-R rates were higher locally than nationally, indicating the need for continued management. The rate of drug resistance among previously treated patients reflects the rationality for treatment strategies at the local level to control the spread of DR-TB strains. In Luoyang City, the resistance rate was significantly higher in patients previously treated for MTB infection than newly diagnosed cases and the detection rates of INH-R, RFP-R, and MDR-TB tended to increase over the 3-year study period (data not shown), suggesting a continued need to strengthen compliance with medication and management strategies. Phenotypic drug susceptibility methods are time-consuming, leading to a dependence on clinical empiric medication, which also can promote acquired resistance. Therefore, faster and more accurate methods are needed to screen for drug susceptibility, especially in developing countries.

The initial and acquired drug resistance rates were both higher in urban than rural areas, indicating that residents of cities are at greater risk for DR-TB due to the relatively higher population density and mobility [28–30]. In addition, the greater availability of healthcare could contribute to the high drug resistance rates in cities. Although the rate of drug resistance was higher in patients with positive vs. negative sputum results, the results of this study showed that the difference in INH-R and MDR detection rates between patients with positive vs. negative sputum results were all not significant. Also, according to the multivariate regression model, the OR value was relatively close to 1 for the association of positive sputum results with an increased risk of drug resistance. Therefore, further studies with larger cohorts are needed to determine whether a positive sputum result is actually a risk factor for drug resistance.

In the multivariate model, history of treatment and younger age were associated with an increased risk for resistance to RFP and INH [31, 32]. Previous studies have found that females are at an increased risk for RFP-R [33]. In contrast, the results of the present study found that males were at an increased risk for RFP-R and INH-R, as well as those aged < 51 years, consistent with the findings of previous studies [33], but inconsistent with the age group of drug resistance in studies conducted in Anhui and Taiwan, China [34, 35]. The higher drug resistance rate in females than males aged 41–45 and 71–75 years may be due to fewer cases of MTB infections among urban males and the relatively high rate among previously treated females in these age groups.

In Luoyang City, the most common genetic mutation associated with RFP-R occurred at codons 529–533 and 521–528, which may be associated with mutations at codon 531 (TCG) and codon 526 (CAC), respectively [36–38]. Consistent with previous reports [39, 40], two mutations, katG315 and the inhA promoter region, were associated with INH-R. These findings suggest that both mutations are potential molecular biomarkers for screening of DR-TB. RFP and INH are key anti-TB drugs, thus resistance is likely to negatively affect patient care and the use of hospital resources. RFP-R is an indicator of the development of MDR-TB. The molecular basis for the spread of MDR-TB is mainly associated with katG315, codon 529–533, codon 521–528, and the inhA promoter region. So, blocking the mechanism of transmission via katG315 and codon 529–533 could hinder the emergence and spread of DR-TB.

The aim of this study was to assess HRM detection and TB resistance of confirmed and suspected cases of TB, which only found that the spread of MTB and drug resistance patterns were associated with high-risk populations, at least to a certain extent. However, data regarding TB transmission and resistance analysis in the region were limited, thereby warranting further studies. Besides, gene sequencing was not conducted to confirm the accuracy of the single nucleotide polymorphisms detected by HRM analysis or multiple mutations associated with drug resistance.

## Conclusion

The majority of resistant strains were isolated from males, who are at a highest risk for MTB infection in adulthood. Therefore, testing of this population is particularly important. The highest rate of MTB infection occurred in males aged 26–30 years and females aged 21–25 years. Prevention and control of MTB infection in rural populations cannot be taken lightly, although urban populations are also at a high risk of MTB infection and high resistance rates. Previous treatment is a risk factor for drug resistance. In addition, mutations to katG315 and codon 529–533 were associated with DR-TB and, thus, should be considered when selecting a treatment strategy. Overall, populations at high risk for MTB infection include females aged < 51 years, males at all ages, individuals previously treated for MTB infection, and those residing in urban areas.

## Data Availability

The datasets used and/or analyzed during the current study available from the corresponding author on reasonable request.
